# Multidrug resistance in cancer: current understandings and future perspective

**DOI:** 10.1186/s43556-026-00502-8

**Published:** 2026-06-26

**Authors:** Yang Qiao, Hongliang Mao, Jianyu Nie, Menghui Liu, Qiyue Lou, Peng Gao, Xingliang Dai

**Affiliations:** 1 Department of Neurosurgery, The First Affiliated Hospital of Anhui Medical University, Hefei, 230022 People’s Republic of China; 2 Department of Neurosurgery, The Second Affiliated Hospital of Soochow University, Suzhou, Jiangsu 215004 People’s Republic of China; 3 Department of Clinical Medicine, The Second Clinical College of Anhui Medical University, Hefei, 230022 People’s Republic of China; 4 Department of Research & Development, East China Institute of Digital Medical Engineering, Shangrao, 334000 People’s Republic of China

**Keywords:** Multidrug resistance, Drug Resistance, Tumor microenvironment, Nanomedicine, Clinical translation, Cellular plasticity

## Abstract

Multidrug resistance (MDR) remains the core clinical barrier limiting the achievement of durable and effective treatment for various tumors. With the deepening of understanding, MDR has been redefined from the traditional, static “drug efflux” model to a systemically adaptive survival strategy driven by highly heterogeneous tumor populations under therapeutic pressure, characterized by dynamic evolution. This complex phenotype arises from the deep intertwining and synergistic action of multi-layered survival networks. Herein, this review systematically delineates the evolution and multidimensional remodeling of the underlying mechanisms of MDR. We comprehensively outline the cell-intrinsic, autonomous resistance mechanisms of tumor cells, including intracellular drug redistribution and evasion of non-apoptotic cell death pathways. Concurrently, we summarize the tumor microenvironment (TME)-mediated, non-autonomous resistance mechanisms, such as physical barriers formed by stromal cells, intercellular communication networks, and the establishment of a profoundly immunosuppressive microenvironment. Building on this foundation, the review critically assesses the current drug resistance dilemmas faced by various therapeutic modalities and refractory cancer types. It focuses on discussing novel, precision reversal strategies targeting MDR, including nanotechnology-based delivery systems, single-cell and spatial omics analysis, and artificial intelligence (AI) large model-driven predictive and interventional systems. Furthermore, this review explores the underlying reasons for the repeated clinical failures of traditional single-target interventions. It outlines a direction for future translational research: a paradigm shift from “singular target killing” to “multidimensional ecological remodeling,” advancing towards a closed-loop, personalized precision therapy framework based on dynamic monitoring and multi-target synergistic intervention.

## Introduction

Tumor multidrug resistance (MDR) is the core clinical challenge responsible for the failure of antitumor therapy, disease recurrence, and even patient death. Traditionally, the understanding of MDR has long been confined to the “drug efflux pump” model, simplifying it as a passive defense mechanism, represented by the overexpression of ABC transporters, aimed at reducing intracellular drug concentration [1]. However, with the rapid advancement of multi-omics technologies, single-cell analysis, and systems oncology, the cognitive boundaries within the field have been profoundly expanded. Modern research has profoundly revealed that MDR is by no means a singular, passive cellular defense, but rather a holistic, dynamically evolving survival adaptation strategy [2].

Under the formidable pressure of therapy, highly heterogeneous tumor populations demonstrate remarkable evolutionary resilience. They achieve self-renewal and therapy escape through genomic instability, epigenetic remodeling, metabolic reprogramming, and the remodeling of the tumor microenvironment (TME) [3]. The current consensus posits that MDR is governed by a deeply intertwined, two-dimensional defense network. One dimension involves cell-intrinsic autonomous adaptations, including the evasion of various programmed cell death pathways (e.g., apoptosis, ferroptosis, cuproptosis), profound epigenetic and metabolic reprogramming, and phenotypic plasticity leading to drug-tolerant persister cells (DTPs) or cancer stem cells (CSCs) states [4, 5]; Another dimension entails non-autonomous protection mediated by the TME, manifested as physical and biochemical barriers established by cancer-associated fibroblasts (CAFs), the formation of immunosuppressive niches, and the “horizontal transmission” of resistant phenotypes via intercellular communication [6, 7]. These two dimensions are not isolated; rather, through continuous and dynamic bidirectional crosstalk, they collaboratively construct a robust and comprehensive drug resistance network.

Despite significant advances in chemotherapy, targeted therapy, and immune checkpoint inhibitors, this synergistic defense network—forged by intrinsic tumor cell plasticity and the extrinsic TME—continues to pose formidable challenges in translating basic mechanistic insights into effective clinical interventions. In this context, emerging combinatorial strategies are demonstrating unprecedented potential. These include targeting vulnerabilities in intrinsic metabolism and cell death checkpoints, systemically reprogramming the immunosuppressive TME, developing nanomedicine-based drug delivery platforms, and implementing AI- and big data-driven dynamic treatment monitoring [8–10]. Collectively, these approaches are paving the way for new therapeutic paradigms capable of overcoming tumor spatiotemporal heterogeneity, predicting evolutionary trajectories, and enabling personalized, precise intervention.

**Fig. 1:**
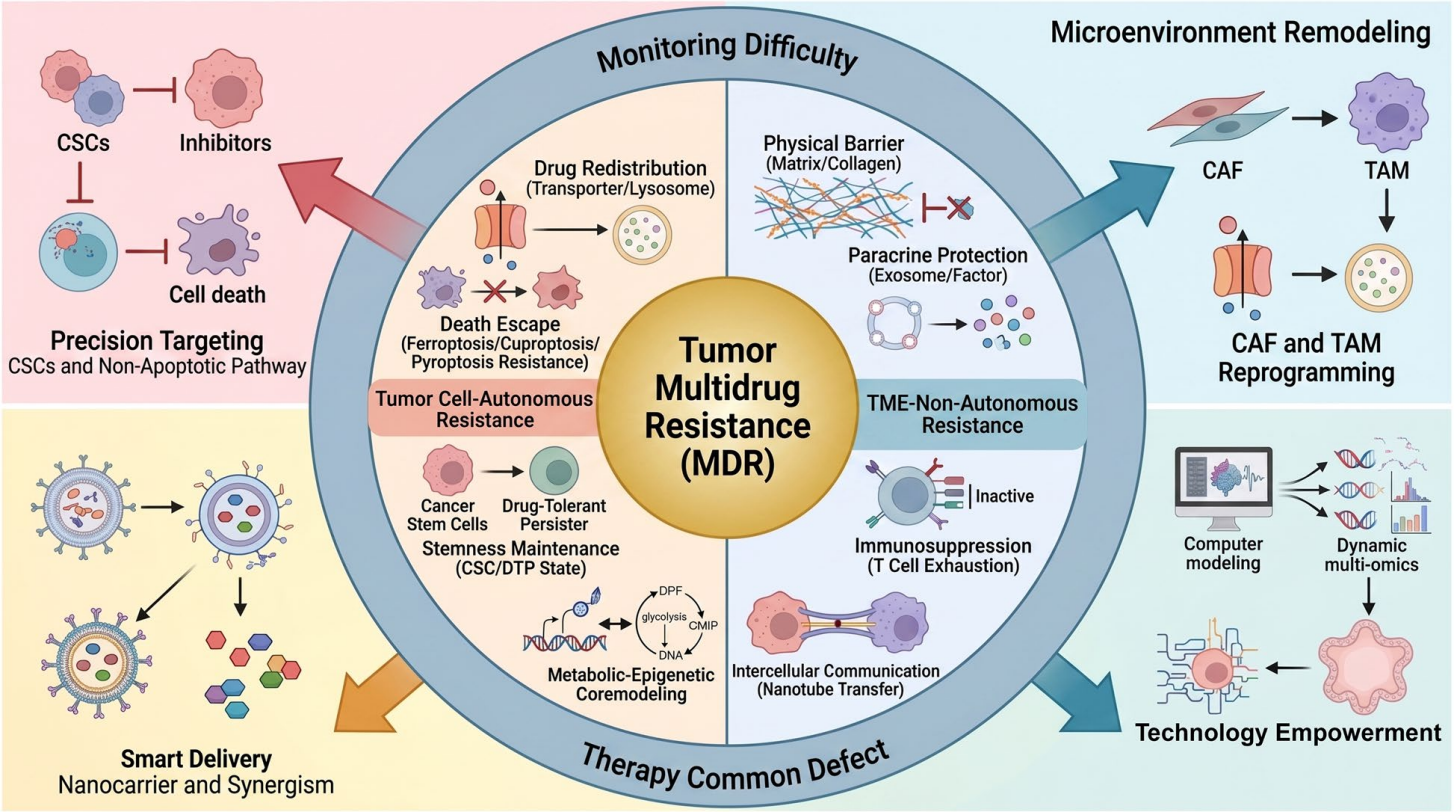
Mechanisms, challenges and therapeutic strategies of tumor MDR.​ This figure provides a comprehensive overview of the multifaceted nature of tumor multidrug resistance, integrating intrinsic cellular mechanisms, dynamic tumor microenvironment interactions, the resulting clinical challenges, and the evolving landscape of reversal strategies. (MDR: Multidrug resistance; TME: Tumor microenvironment; CSC: Cancer stem cell; DTP: Drug-tolerant persister; CAF: Cancer-associated fibroblast; TAM: Tumor-associated macrophage)

Against this evolving conceptual landscape, this Review systematically delineates the multidimensional regulatory network underpinning cancer MDR (Fig. 1). Moving beyond static definitions, we categorize MDR into four evolutionary subtypes​ that capture the continuum from transient adaptation to fixed genetic resistance. Central to this framework is the deconstruction of a dual-layered defense architecture: the integration of cell-intrinsic autonomous mechanisms—encompassing metabolic-epigenetic rewiring and non-apoptotic cell death evasion—with TME-mediated non-autonomous mechanisms, including physical barriers and immunosuppressive niches. We subsequently evaluate the clinical dilemmas imposed by MDR across major therapeutic modalities and refractory cancer types, highlighting critical translational gaps in current monitoring strategies. Finally, we explore emerging precision reversal strategies converging at the intersection of nanomedicine, artificial intelligence, and ecological remodeling. By providing a systematic framework and a forward-looking translational perspective, this review seeks to contribute to the development of effective strategies for dismantling the tumor MDR ecosystem and achieving long-term disease control.

## Definition and evolutionary development of MDR

The definition of MDR has continuously evolved alongside the expansion of our conceptual understanding. Traditionally, it was narrowly conceptualized as the cross-resistance of tumor cells to other drugs with divergent structures and mechanisms following exposure to a single anti-tumor agent, with its theoretical underpinning primarily focused on the “efflux pump effect” centered on the overexpression of the ABC transporter family [11]. Contemporary research increasingly views it as a global, dynamically evolving survival adaptation strategy. It represents the self-evolution of highly heterogeneous tumor cells, achieved through the deep interplay of multiple mechanisms under therapeutic selective pressure [12]. Its connotation has now extended beyond the mere maintenance of intracellular drug concentration, encompassing the systemic evasion and remodeling of both the tumor itself and its external microenvironment [13]. From this perspective, MDR manifests biologically and clinically as four highly intertwined evolutionary subtypes: Firstly, de novo(primary) resistance, where tumors inherently harbor resistance mutations or exist in a profoundly immunosuppressed state, exhibiting absolute non-response from the initial treatment phase [14]; Secondly, adaptive resistance, referring to the rapid phenotypic switching and survival maintenance achieved by tumors in the very early stages of drug exposure via non-mutation-dependent signaling pathway rewiring or transcriptional reprogramming [15]; Thirdly, *acquired* resistance, which is essentially clonal evolution under protracted Darwinian selection pressure, involving the enrichment of resistant subclones within minimal residual disease or clinical relapse driven by concomitant de novo driver alterations [16]; Fourthly, cross-resistance, where exposure to a single therapeutic pressure activates underlying anti-apoptotic pathways, leading to spontaneous resistance to heterogeneous therapies not previously encountered [17, 18]. These four subtypes are not isolated or discrete but constitute a spatiotemporally overlapping continuum. De novo resistance lays the foundation for the tumor's adaptive remodeling; adaptive resistance acts as a transitional buffer against acute drug toxicity, buying the “evolutionary window” necessary for the accumulation and clonal expansion of mutations conferring acquired resistance; and cross-resistance represents the culmination of this sequence of events at the terminal stage of therapy [12]. Collectively, they weave the multidimensional defensive network by which tumors counteract systemic eradication.

## Intrinsic autonomous MDR-driven mechanisms in tumor cells

Multidrug resistance originates from the intrinsic adaptability of tumor cells. Beyond external influences, cancer cells possess an autonomous defense arsenal enabling survival against therapy. This section outlines the intrinsic mechanisms driving MDR, including the regulation of intracellular drug distribution, rewiring of oncogenic pathways, activation of alternative survival routes, and reprogramming of epigenetic and metabolic networks. We also examine how tumor cell plasticity—exemplified by CSCs and DTP cells—integrates these mechanisms into a unified resistance continuum. Deciphering these internal strategies is essential for understanding the broader resistance ecosystem.

### Intracellular drug redistribution

Tumor cells achieve MDR through the precise regulation of intracellular drug distribution and concentration—a fundamental autonomous defense mechanism. While enhanced drug efflux, epitomized by P-glycoprotein (P-gp/ABCB1) overexpression, has long been considered the classic paradigm of MDR, clinical strategies based on the simple competitive inhibition of this target have repeatedly faltered [19, 20]. This failure has thus spurred a deeper, more systemic inquiry.

**Fig. 2:**
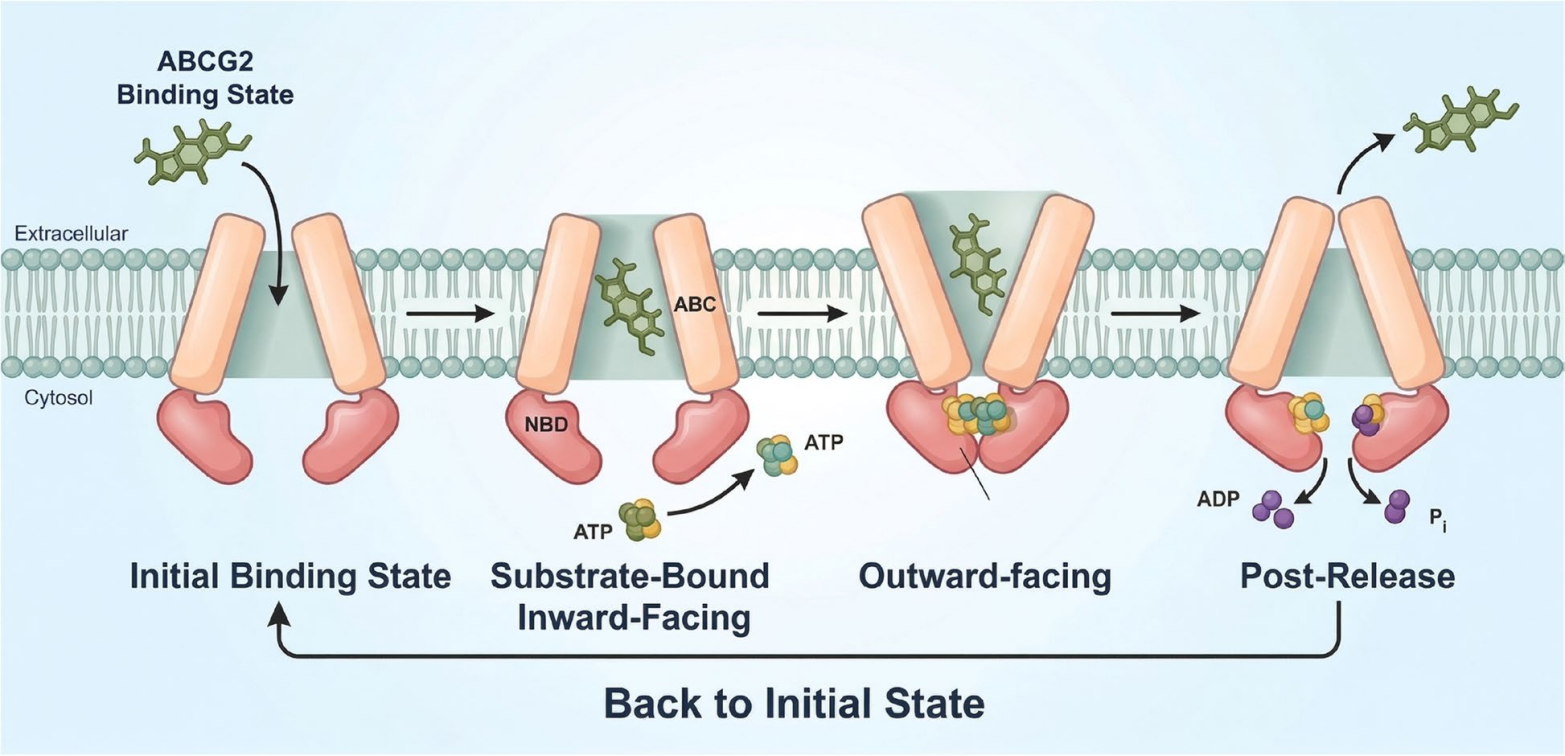
Schematic representation of the ABCG2 transporter substrate transport cycle.​ ABCG2 mediates multidrug resistance through an ATP-dependent cyclic mechanism involving substrate recognition, high-affinity binding, ATP-induced conformational switching, and subsequent drug extrusion into the extracellular space. (ABCG2: ATP-binding cassette subfamily G member 2; NBD: Nucleotide-binding domain; ATP: Adenosine triphosphate; ADP: Adenosine diphosphate; Pi: Inorganic phosphate.)

Research into drug efflux mechanisms now extends far beyond cataloguing transporter function, probing the atomic-level conformational dynamics and intricate regulation of these molecular pumps. Advances in structural biology, particularly cryo-electron microscopy, have resolved the conformational transitions of ABC transporters during their catalytic cycle. These studies reveal sophisticated allosteric communication between nucleotide-binding domains and substrate-binding pockets [21], providing a structural blueprint for novel therapeutic strategies such as allosteric modulators [11]. This foundational knowledge is powerfully exemplified by studies on ATP-binding cassette subfamily G member 2 (ABCG2), a transporter of high clinical relevance due to its expression in protective barriers and CSCs. Structural insights delineate its transport mechanism, wherein substrates bind in an initial hydrophobic pocket (Cavity 1) before being extruded extracellularly via a conformational shift to a release pocket (Cavity 2) driven by ATP hydrolysis [22]. The activity of ABCG2 is governed by a complex regulatory network involving epigenetic modifications, transcription factors responsive to hypoxia and xenobiotics, and extensive crosstalk with pro-survival pathways like PI3K/Akt. In refractory cancers such as glioblastoma, lung, and breast cancer, ABCG2 overexpression is not only a direct mediator of resistance to agents like topotecan and mitoxantrone but also a marker of stemness associated with tumor recurrence (Fig. 2) [23–26].

In parallel, the mechanisms governing drug “influx” and intracellular “sequestration” have undergone significant conceptual revision. The role of solute carrier (SLC) transporters in mediating drug uptake is now recognized as equally critical. Resistance arising from impaired influx is understood to involve not only transcriptional downregulation but also post-translational mechanisms; for instance, specific deubiquitinases can induce resistance by promoting the degradation of SLC transporter proteins [27, 28]. Following cellular entry, the sequestration of drugs within organelles like lysosomes constitutes an active resistance process [29]. Moving beyond the passive model of “ion trapping” in acidic compartments, strategies to reverse drug protonation have emerged to disrupt this barrier [30]. Furthermore, epigenetic regulators are now known to directly control the fate of sequestered drugs. Inhibitors of type I protein arginine methyltransferases (PRMTs), for example, can chemo-sensitize tumors by blocking lysosomal exocytosis, thereby trapping drugs inside the cell [31].

In summary, the contemporary understanding of intracellular drug redistribution has evolved from a static, transporter-centric model to a dynamic and integrated view of a complex regulatory system. This system encompasses the dynamic structures and interactions of ABC and SLC transporters, multifaceted regulatory networks involving processes such as ubiquitination and epigenetic modification, and the coordinated functions of organelles like lysosomes.

### Abnormal drug targets and compensatory activation of signaling pathways

The rewiring of oncogenic signaling pathways represents a fundamental adaptive mechanism underlying tumor MDR. This process is often initiated by aberrant alterations in the drug targets themselves, which trigger complex compensatory activation networks. The most direct form involves genetic or epigenetic changes in the target, known as “on-target” alterations [32, 33]. Even when targeting novel therapeutic entities like the WRN helicase, tumors can develop specific resistance through acquired mutations in the WRNgene itself [34]. Beyond these direct changes, a more prevalent and complex clinical challenge is the dynamic “rewiring” and compensatory activation of parallel signaling networks. When a primary oncogenic driver pathway is effectively blocked, tumor cells can activate alternative signaling routes to achieve a survival “bypass.” A key example is the aberrant activation of the Syndecan-1 signaling axis, which serves as a critical alternative survival pathway following inhibition of KRASG12C [35]. Such compensatory signaling often converges on central hub proteins. For instance, SHP2 acts as a key node, and its inhibition can reverse cross-resistance mediated by the reprogramming of various receptor tyrosine kinases [36]. Furthermore, the stabilization of the resistant phenotype frequently involves self-reinforcing regulatory circuits, such as a positive feedback loop between c-Src and the long non-coding RNA *LIST*, which continuously drives pro-survival signals to entrench the resistant state [37]. At a more dynamic level, the profound signaling plasticity of tumor cells underpins this rapid adaptive capability. Therapeutic pressure can induce a plastic cellular state, permitting transient drug tolerance through rapid, albeit initially unstable, network rewiring [38, 39]. This adaptive process can be conceptualized as a form of network “learning,” wherein tumor cells dynamically diminish their reliance on the inhibited primary pathway while simultaneously reinforcing or establishing alternative pathways, ultimately leading to a stable resistant memory. Genomic instability acts as a critical catalyst in this process, dramatically accelerating the emergence of adaptive mechanisms described above [40]. To preempt such evolving resistance, emerging systems biology approaches—such as analyses based on “genetic contextual drift”—can offer promising strategies for prospectively identifying and targeting these dynamically emerging vulnerabilities [41].

### Activation of cellular survival pathways

The activation and remodeling of pro-survival signaling pathways constitute a robust, cell-intrinsic foundation for MDR in tumors, creating a powerful integrated network that suppresses cell death and promotes survival. Core pathways such as PI3K/AKT/mTOR and MAPK are central hubs in this network. Resistance mechanisms involving these pathways have evolved beyond simple acquired mutations to encompass complex feedback rewiring and reprogramming of cellular states [42, 43]. This is exemplified in PIK3CA-mutant breast cancer, where overcoming resistance to CDK4/6 inhibitors and endocrine therapy requires the concurrent targeting of both PI3K and mTOR signaling nodes, underscoring the significant redundancy within this pathway [44]. In MAPK-driven tumors, resistance demonstrates profound adaptive plasticity. For example, melanomas can rewire dependency from BRAF to RAF1 by upregulating phosphodiesterase PDE4D [45], or generate pro-survival protein isoforms through aberrant, genome-wide alternative splicing [46]; Similarly, in colorectal cancer, MAPK signaling can induce a plastic transition of epithelial cells to a regenerative stem cell-like state, thereby directly driving therapy resistance [47]. Parallel to these, the JAK-STAT pathway—which integrates signals from inflammation, immunity, and stemness—plays a prominent role in stabilizing resistant phenotypes. In inflammatory breast cancer, it is instrumental in driving a chemotherapy-tolerant, stem-like cell state [48], The therapeutic potential of targeting such survival networks is highlighted by agents like the multi-target inhibitor Lestaurtinib, which demonstrates efficacy against both therapy-sensitive and -resistant ovarian cancers by converging on key nodes including JAK/STAT [49]. Furthermore, the NF-κB pathway functions as a critical integrative center for multidimensional resistance. Its inhibition is therefore considered a promising strategy to overcome broad-spectrum resistance [50], This is evident in lymphoma, where the TME can mediate resistance to BH3 mimetics by activating the non-canonical NF-κB pathway [51]. In summary, tumor cells establish a remarkably stable and adaptive MDR network not through the isolated action of single pathways, but via the dynamic interweaving and remodeling of multiple core survival circuits, including PI3K/AKT/mTOR, MAPK, JAK-STAT, and NF-κB.

### Non-apoptotic cell death pathways

Under sustained evolutionary pressure, the survival strategy of tumor cells has progressed beyond simple apoptosis evasion to encompass the construction of a sophisticated, multi-layered defense network designed to systematically circumvent various forms of non-apoptotic programmed cell death, including ferroptosis, cuproptosis, pyroptosis, and necroptosis.

Resistance to ferroptosis involves establishing a multi-tiered defensive system that ranges from altering membrane composition to reinforcing antioxidant pathways [52]. The first line of defense involves the physical remodeling of the plasma membrane. By altering lipid composition—for example, through the upregulation of specific sterols like 7-dehydrocholesterol—tumor cells reduce the membrane's inherent susceptibility to lipid peroxidation [53]. Concurrently, the core glutathione-dependent antioxidant machinery is strengthened through multiple regulatory layers. Transcriptional and post-transcriptional mechanisms, mediated by factors such as HMGA1 or the long non-coding RNA Linc01833, upregulate the cystine transporter SLC7A11 to secure the supply of glutathione precursors [54]; At the post-translational level, peroxiredoxin 6 (PRDX6) can act as a molecular chaperone, complexing with GPX4 to promote its membrane localization and enhance its activity, thereby creating a coupled “reduction–hydrolysis” repair system [55]. Furthermore, specific cellular states and organelle functions are co-opted for structural resistance. CSCs, for instance, downregulate PCK2 to alter membrane phosphatidylethanolamine composition and resist ferroptosis [56]; Lysosomes also serve as key defensive components in tumors with hyperactive AKT signaling, actively expelling accumulated lipid peroxides via TRPML1 channel-mediated exocytosis [57]. Crucially, this intrinsic ferroptosis defense is profoundly augmented by the TME. Tumor-associated macrophages (TAMs) can deliver PRDX6 to inhibit mitophagy and indirectly bolster ferroptosis resistance [58]; while adipocyte-secreted 3-hydroxykynurenine can inhibit ferritinophagy to provide protection [59].

Resistance to the more recently characterized process of cuproptosis often leverages the tumor’s inherent pathophysiology. Tumor cells, especially within solid tumors, exploit their hypoxic niche as a fundamental protective mechanism [60]. Here, hypoxia-inducible factor 1-alpha (HIF-1α) emerges as a central regulator, dismantling cuproptosis through a dual strategy: by upregulating copper-chelating metallothioneins and by metabolically reprogramming cells to downregulate DLAT, a key enzyme in the lipoylation pathway essential for cuproptosis execution [52, 61]. In parallel, oncogenic signaling cascades can directly inhibit the process. In triple-negative breast cancer, for example, AKT1 phosphorylates and inactivates FDX1, a core cuproptosis protein, to confer resistance [62].

The evasion of inflammatory cell death pathways—pyroptosis and necroptosis, which are linked to anti-tumor immunity—involves particularly sophisticated and subversive strategies [63, 64]. Tumor cells manipulate complex epitranscriptomic networks to inhibit these pathways. One demonstrated mechanism involves the circular RNA circPDIA3, which sequesters miR-449a to establish a positive feedback loop that ultimately suppresses the palmitoylation and pore-forming activity of the pyroptosis executor GSDME [65]. Perhaps even more remarkably, the necroptosis pathway itself can be co-opted to foster an immunosuppressive milieu. Research indicates that chemotherapy can preferentially activate PARP1 in tumor-associated endothelial cells. PARP1 then induces necroptosis in these cells via PARylation of MLKL. The ensuing death of endothelial cells results in a “paracrine vascular” release of factors like SDF1, which suppresses cytotoxic T-cell infiltration and thereby drives both immunosuppression and therapy resistance [66].

### Epigenetic and transcriptional reprogramming

Continuous therapeutic pressure drives profound epigenetic reprogramming in tumor cells. They dynamically regulate genome accessibility by globally rewriting “epigenetic codes,” such as histone modifications and DNA methylation, and remodeling the three-dimensional chromatin architecture. This process dynamically orchestrates the activation of powerful pro-survival transcriptional programs and “locks” the acquired drug-resistant phenotype into cellular memory, enabling its intergenerational inheritance [67, 68]. This reprogramming occurs at multiple levels. At the chromatin regulatory level, signaling pathways can drive the localization of non-canonical proteins to chromatin, where they recruit remodeling complexes to directly mediate resistance programming at the epigenetic level. For example, mTORC2 drives the recruitment of the SWI/SNF complex to chromatin by cGAS to promote chemotherapy resistance in colorectal cancer [69]. Concurrently, novel histone modifications are continually being identified as key regulatory mechanisms. For instance, lactate-induced histone lactylation can reprogram the tumor immune microenvironment [70], while dysregulation of histone crotonylation levels is directly linked to EGFR-TKI resistance in lung cancer, suggesting the potential to reverse resistance by restoring specific modifications [71]. More importantly, the core output of these epigenetic changes is to confer robust transcriptional plasticity to tumor cells. Transcription factors like SOX9 can drive the establishment of a stem cell-like transcriptional state, directly leading to platinum resistance in ovarian cancer [72]; Lineage-tracing evidence based on organoids further reveals that this epigenetically mediated plasticity can drive the generation of highly heterogeneous resistant clonal populations in a “one-to-many” pattern [5]. However, drug resistance is not insurmountable. While epigenetic remodeling confers a survival advantage, it simultaneously exposes new molecular vulnerabilities. For example, in castration-resistant prostate cancer, EZH2 drives oncogenic translation through a non-canonical function, leading to enzalutamide resistance while also creating a “synthetic lethal” sensitivity to translation inhibitors [73, 74]. This suggests that targeting epigenetic regulators themselves and designing rational combination therapies constitute effective strategies for overcoming clinical drug resistance.

### Tumor cell plasticity: CSCs and DTPs

The conceptual framework for MDR has evolved significantly, moving beyond the classic static clonal selection model toward a redefinition of MDR as a dynamic adaptive process fundamentally driven by tumor cell plasticity. Central to this concept is the “resistance continuum,” where CSCs and DTPs represent not fixed endpoints, but fluid phenotypic states connected by bidirectional transitions [12, 75].

While both populations contribute to therapeutic failure, their epigenetic landscapes and functional attributes are distinct. CSCs are defined by high transcriptional activity and self-renewal capacity, marked by elevated levels of H3K4me3 and H3K27ac at stemness-related enhancers [76]. In contrast, DTPs are characterized by a profound proliferative arrest and a reliance on H4K20me3-mediated heterochromatin compaction to deeply silence inflammation-related genes, thereby evading immune surveillance [77, 78]. This epigenetic divergence is functionally mirrored by metabolic rewiring; for instance, glioblastoma stem cells hijack the circadian clock to regulate copper homeostasis against cuproptosis [79], whereas DTPs upregulate cholesterol synthesis via PRDM9 to create a metabolic sanctuary against oxidative stress [80].

Crucially, the transition between these states is reversible, a feature empirically validated by lineage-tracing studies. Under acute therapeutic pressure, CSCs can shed their stemness markers to enter the transient, drug-tolerant DTP state—acting as a critical “incubator” for survival [81]. Conversely, as drug pressure subsides or microenvironmental cues shift, DTPs can reactivate stemness programs and re-enter the cell cycle, giving rise to relapsed tumors [82]. This reversible plasticity implies that targeting either state in isolation is insufficient. Indeed, emerging strategies aim to eradicate the DTP reservoir to prevent clonal outgrowth; for example, CAR-T cells targeting TROP2—a surface antigen highly expressed on DTPs—have demonstrated efficacy in depleting these resistant seeds in EGFR-mutant non-small cell lung cancer [83].

In summary, tumor cell plasticity weaves a continuous defensive network by linking the intrinsic resilience of CSCs with the transitional, adaptive niche of DTPs. Targeting the unique epigenetic and metabolic dependencies of these plastic states is paramount for achieving durable disease control.

### Metabolic reprogramming and redox homeostasis imbalance

Therapeutic pressure drives tumor cells to reprogram their metabolic and redox networks, enabling them to acquire critical survival resources. Notably, metabolites that accumulate during this reprogramming can themselves function as regulatory signals, influencing downstream processes such as signal transduction, epigenetic regulation, and antioxidant responses. This synergy creates a multi-layered and integrated defense network that underpins drug resistance [84].

A hallmark of this adaptation is the remarkable metabolic plasticity of resistant cells. They often maintain a reliance on oxidative phosphorylation (OXPHOS), which serves as a fundamental energetic anchor supporting both drug resistance and metastatic potential [85], Furthermore, these cells can bypass targeted therapies through the dynamic rerouting of metabolic pathways. A key example is the remodeling of the transsulfuration pathway observed under sustained inhibition of BRAFV600E [86]. Beyond fueling biosynthesis, accumulated metabolic intermediates directly contribute to resistance as signaling molecules and donors for epigenetic modifications, establishing a functional “metabolism–epigenetics” axis. For instance, lactate-driven lactylation of the NBS1 protein enhances DNA damage repair efficiency, thereby conferring profound tolerance to chemotherapy [87].

Concurrently, to survive the lethal oxidative stress induced by radio- and chemotherapy, tumor cells robustly reinforce their antioxidant defenses. A central player in this response is the transcription factor NRF2. In head and neck squamous cell carcinoma, constitutive activation of NRF2 signaling not only drives extreme radiotherapy resistance but also profoundly suppresses anti-tumor immunity [88]. Similarly, in glioma, tumor cells escape radiotherapy-induced ferroptosis by upregulating GDF15 to antagonize lipid peroxidation, while simultaneously reshaping the immune microenvironment to block cytotoxic responses [89]. Critically, this reprogramming-induced redox imbalance extends its influence beyond the tumor cell, profoundly reshaping the TME through bidirectional crosstalk [90]. Environmental stressors within the TME, such as hemorrhage, can activate the NRF2 pathway in TAMs, promoting tumor progression and immunotherapy resistance [91]; Conversely, therapeutic interventions can exploit this interconnectivity. Targeting intrinsic tumor cell nodes—such as the tyrosine phosphatase SHP-1 in leukemia stem cells—can reverse the immunosuppressive TME state and re-sensitize tumors to chemotherapy by inducing specific metabolic changes [92]. In summary, metabolic reprogramming and the disruption of redox homeostasis systematically elevate the survival threshold of tumor cells.

### Cross-regulatory network of intrinsic cellular MDR

**Fig. 3:**
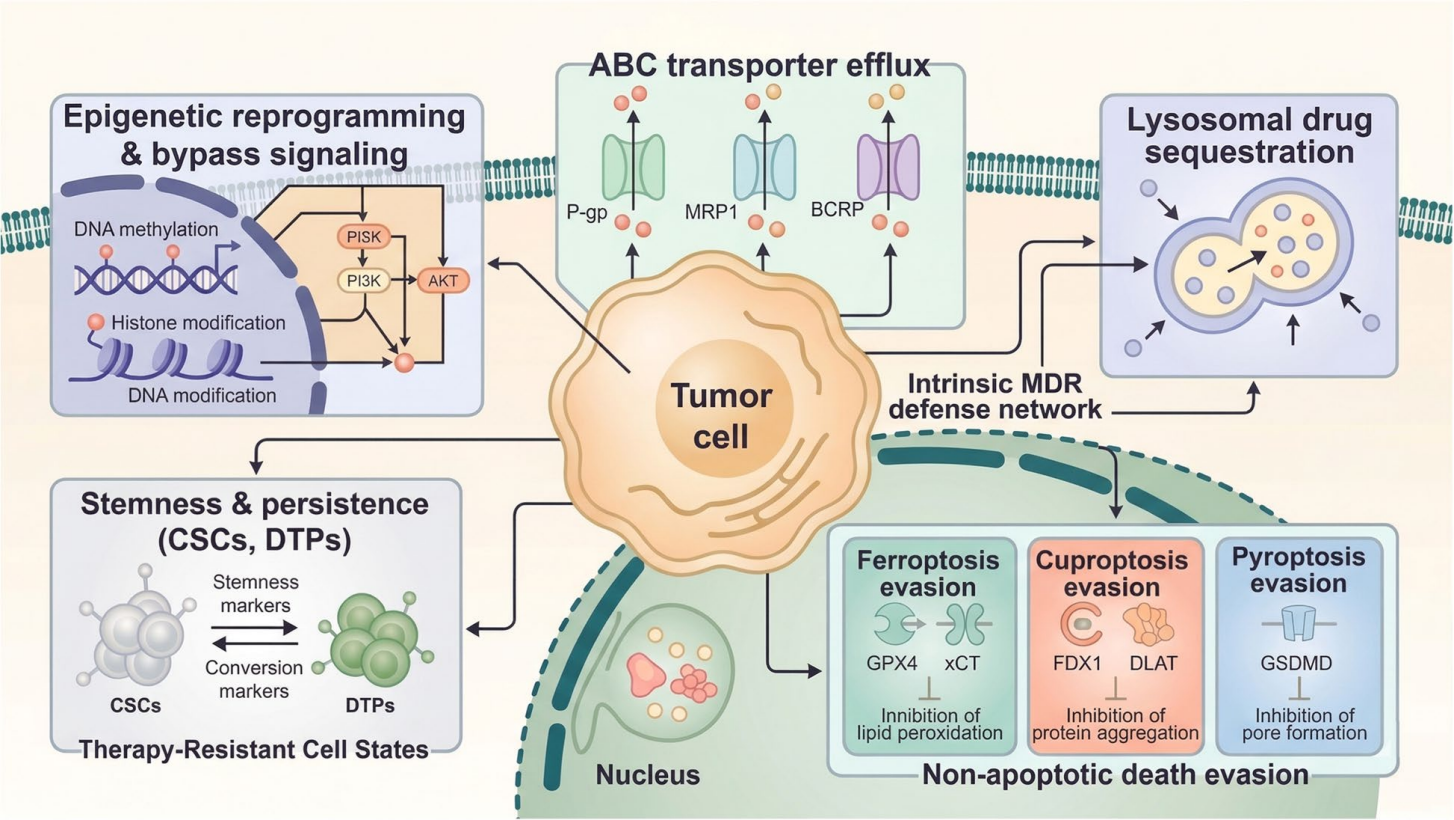
The intrinsic MDR defense network of tumor cells.​ An integrated intracellular network orchestrates multidrug resistance through coordinated drug efflux, metabolic reprogramming, DNA damage repair enhancement, apoptosis evasion, and epigenetic or transcriptional rewiring. (ABC: ATP-binding cassette; DTP: Drug-tolerant persister; GPX4: Glutathione peroxidase 4; FDX1: Ferredoxin 1; DLAT: Dihydrolipoamide S-acetyltransferase; GSDMD: Gasdermin D)

In summary, intrinsic MDR in tumor cells constitutes a complex adaptive system forged by a dynamic and highly interconnected regulatory network (Fig. 3). Its defining hallmark is the profound crosstalk and functional integration among distinct resistance mechanisms. This integration is exemplified by the cooperative relationship between dysregulated drug disposition (e.g., via ABC/SLC transporters) and extensive epigenetic reprogramming (e.g., histone modifications), which together precisely orchestrate the intracellular fate of therapeutics. Furthermore, target abnormalities and compensatory signaling (e.g., “on-target” mutations, bypass pathway activation) rapidly initiate the global rewiring and potentiation of key pro-survival pathways (e.g., PI3K/AKT/mTOR, JAK-STAT), erecting a robust anti-apoptotic barrier. Concurrently, to evade emerging forms of programmed cell death—such as ferroptosis, cuproptosis, and pyroptosis—tumor cells must undergo extensive metabolic and redox network restructuring. This restructuring provides necessary metabolites and antioxidant defenses; yet, the resulting metabolic alterations also function reciprocally as signaling molecules that directly fuel epigenetic and transcriptional reprogramming, thereby locking in the resistant phenotype. Ultimately, these interlinked processes—encompassing drug handling, signaling adaptation, death evasion, and metabolic–epigenetic rewiring—converge to define and sustain the core plasticity of tumor cells (e.g., CSCs and DTPs). This integrated network enables their dynamic evolution along a phenotypic “resistance continuum,” thereby encapsulating the fundamental architecture of cell-intrinsic MDR.

## TME-mediated non-autonomous MDR mechanisms

While intrinsic mechanisms provide a primary defense, the TME serves as a non-cell-autonomous collaborator that shapes the resistance phenotype. Therapeutic pressure selects for resistant clones while remodeling the stroma to create a protective niche shielding tumor cells from drugs and immune surveillance. Moving beyond the cancer cell, this section examines how physical barriers—such as the extracellular matrix and CAFs—restrict drug penetration. We further explore stromal-derived paracrine networks, immunosuppressive microenvironments, and intercellular communication via tunneling nanotubes and exosomes in propagating resistance. Finally, we address how therapies can inadvertently promote the evolution of a more refractory TME.

### Physical barriers of the TME

The physical barriers within the TME, especially the dynamically remodeled network of the extracellular matrix (ECM), play a crucial role in non-autonomous tumor MDR. These physical structures are not merely static obstacles that impede drug penetration and immune cell infiltration; rather, they function as active signaling hubs. Through their mechanical properties (e.g., stiffness, pressure), they actively initiate and sustain aberrant mechano-biochemical signaling crosstalk between tumor cells and stromal cells [93–95].

Specifically, the physical attributes of the ECM are fundamental to its biological function. Studies show that matrix stiffness alone can directly drive resistance to chemotherapeutic drugs by activating mechanosensors (e.g., CD44) on the surface of tumor cells [96]. Furthermore, such mechanical signals can remodel the entire TME's metabolism. In colorectal cancer liver metastasis, for instance, increased matrix stiffness can trigger a metabolic reprogramming crosstalk centered on lipids between tumor cells and CAFs, thereby mediating resistance to anti-angiogenic agents [97].

More importantly, the construction of this physical barrier is a dynamic process orchestrated collaboratively by tumor cells and stromal cells, particularly CAFs. On one hand, tumor cells can actively recruit and “educate” CAFs. Esophageal carcinoma cells, for example, can induce the functional polarization of CAFs by secreting the alarmin S100A8, which activates the CD147 receptor on CAFs, thereby fostering a protective, drug-resistant microenvironment [98]. On the other hand, activated CAFs not only deposit and cross-link vast amounts of ECM but can also exert active mechanical compression forces to directly modulate mechano-signaling in cancer cells, providing them with mechanical cues to adapt to therapeutic pressure [99].

Notably, tumor cells themselves are deeply involved in barrier construction. In non-small cell lung cancer, cancer cells can directly express collagen, forming a physical barrier that not only hinders the infiltration of cytotoxic T cells but also directly leads to acquired resistance to anti-PD-1/PD-L1 immunotherapy [100]. Additionally, CAFs can further consolidate this interwoven physical-biochemical defense network through non-contact means, such as the release of extracellular vesicles that remotely deliver resistance-conferring molecules like proteins and RNAs [101]. Therefore, the key to TME physical barrier-mediated, non-autonomous MDR lies in the deep integration of mechanical properties and biochemical signaling networks [102].

### Paracrine resistance mediated by stromal cells

Within the TME ecosystem that underpins MDR, cancer cells do not endure therapeutic pressure in isolation. Instead, they orchestrate a strategy of non-autonomous evasion by actively reprogramming stromal cells—notably CAFs and TAMs —to construct a resilient, multidimensional “paracrine protective network.” The establishment and maintenance of this network depend critically on intercellular communication mediated by extracellular vesicles (EVs) and soluble factors.

Activated CAFs, for example, secrete EVs capable of mediating broad-spectrum therapy resistance [101]. A specific mechanistic instance involves a distinct CAF subset that releases exosomes enriched with the chaperonin subunit CCT6A. These vesicles directly target and activate the pro-survival β-catenin pathway in gastric cancer cells, conferring profound chemotherapy tolerance [103]. Beyond vesicular signaling, specific stromal subpopulations such as THBS2⁺ CAFs promote resistance through the paracrine secretion of factors like COL8A1, which can induce epithelial-mesenchymal transition (EMT) and activate the PI3K/AKT axis in tumor cells, thereby blocking the cytotoxicity of agents such as oxaliplatin [104]. The functional versatility of CAFs is further demonstrated by their ability to drive microenvironmental resistance through the upregulation of other pathways, including those involving G0S2 and SRC kinase [105, 106]. The complexity of this paracrine network is heightened by its intricate intertwinement with the metabolic and biophysical properties of the TME. For instance, abnormally high matrix stiffness can induce a paracrine lipid metabolic crosstalk between stromal and cancer cells, enabling tumors to circumvent the effects of anti-angiogenic therapies [97].

Acting in concert with CAFs, TAMs constitute another core pathogenic component within this network. TAMs contribute to resistance not only by driving acquired MDR through high expression of enzymes like ADAR1 [107] but also by mediating profound immunotherapy resistance via aberrant sphingolipid synthesis metabolism [108]. Crucially, the tumor-stroma symbiosis underpinning this network is highly dynamic and bidirectional. Under chemotherapeutic stress, cancer cells can actively initiate the dialogue by releasing alarmins such as S100A8 to “educate” and activate CAFs, which in turn construct a reciprocal protective barrier [98]. This reciprocal reprogramming extends to metastatic sites, where colonizing cancer cells induce the formation of metastasis-associated CAFs in distant organs. These educated CAFs can then utilize paracrine signaling to force cancer cells into an autophagy-dependent “dormant” state, thereby shielding them from elimination by classical chemotherapeutic agents [109]. In summary, MDR within the TME is sustained by a sophisticated biochemical scaffold: the paracrine network. Woven from EVs, metabolites, and an array of reprogramming factors, this network constitutes a fundamental non-autonomous pillar of therapeutic resistance.

### Immunosuppressive microenvironment

The immunosuppressive TME serves as a critical permissive niche and a breeding ground for acquired resistance to cancer immunotherapies. Within this network, myeloid and stromal cells function as a coordinated “command center,” with myeloid lineages—particularly TAMs and neutrophils—playing a pivotal orchestrating role. A key mechanism of suppression involves their ability to directly subvert adaptive immunity. Through antigen presentation, these cells can drive the irreversible differentiation of CD8⁺ T cells from a renewable progenitor-exhausted state into a dysfunctional, terminally exhausted state, thereby dismantling cytotoxic anti-tumor responses [110]; Moreover, specific molecular targets on myeloid cells, such as the immunosuppressive receptor CD300ld on neutrophils, have been identified as core regulators of their pro-tumor functions, revealing novel targets for counteracting myeloid-mediated immunosuppression [111]. This suppressive activity is not diffuse but exhibits precise spatial organization. For instance, in ovarian cancer following neoadjuvant chemotherapy, treatment induces the formation of localized niches enriched with specific myeloid subsets that focally drive T cell exhaustion, creating discrete pockets of resistance [112]. Beyond driving active immune suppression, the TME concurrently reinforces an immune-excluded state by constructing multidimensional physical and biochemical barriers [113]. In triple-negative breast cancer, for example, stimulation by sensory neurons induces CAFs to deposit a dense extracellular matrix, forming a physical barrier that impedes cytotoxic T lymphocyte infiltration [114]. Importantly, therapeutic intervention itself can inadvertently fortify this defensive architecture [115]. Prolonged use of EGFR/ALK tyrosine kinase inhibitors in non-small cell lung cancer, for instance, can reprogram the local immune milieu, polarizing macrophages toward an immunosuppressive M2 phenotype and upregulating barrier proteins, which collectively impair T cell function [116]. Tumor cells are also active participants in defense, deploying strategies such as the release of neutrophil extracellular trap (NET)-associated DNA to drive EMT in a CCDC25-dependent manner, thereby broadly compromising therapeutic efficacy [117]. Therefore, effectively reversing immunosuppressive TME-mediated multidrug resistance requires a paradigm shift—from targeting isolated molecular nodes to systemically remodeling the entire network. A promising illustration of this approach is found in castration-resistant prostate cancer, where pharmacological inhibition of PKMYT1 activates the cGAS–STING pathway, converting an “immune-cold” tumor into an “immune-hot” state and thereby sensitizing it to anti–PD-L1 therapy [118]. Consequently, future therapeutic frameworks must integrate combinatorial strategies designed to concurrently modulate myeloid cell function, dismantle physical and molecular barriers, and reverse the dysfunctional differentiation trajectory of T cells within the TME.

### Intercellular communication-mediated transfer of drug-resistant phenotypes

The horizontal propagation and stabilization of MDR within a tumor population critically depend on intercellular communication networks. This non-autonomous transmission is mediated primarily by two core structures: EVs, particularly exosomes, and tunneling nanotubes (TNTs) [119, 120].

Exosomes function as versatile molecular packages, enabling drug-resistant or stromal cells to transfer functional resistance programs to therapy-sensitive neighboring cells. A prime example involves CSCs in non-small cell lung cancer, which secrete exosomes carrying the phosphorylated metabolic enzyme pY105-PKM2. Upon uptake by recipient cells, this cargo induces metabolic reprogramming, confers stem-like properties, and promotes chemotherapy resistance [121]; Beyond influencing other tumor cells, exosomal communication can directly undermine anti-tumor immunity. In head and neck squamous carcinoma, tumor-derived exosomes enriched with the proteasome activator PA28γ induce T cell exhaustion, thereby facilitating immune evasion [122].

For the exchange of larger cargo, a more direct and efficient mode of communication is provided by TNTs. These delicate, filamentous membrane bridges facilitate the intercellular transfer of entire organelles [123]. A striking example of this mechanism is observed in EGFR-mutant lung cancer under targeted therapy pressure, where cancer cells actively “offload” dysfunctional mitochondria to surrounding cancer-associated fibroblasts via TNTs. This disposal mechanism allows tumor cells to clear metabolic waste, sustain survival, and establish a drug-resistant state [124].

Intercellular communication can also contribute to a form of systemic physical defense. For instance, tumor-derived small EVs can adsorb therapeutic nanoparticles, promoting their hepatic clearance and thereby systemically diminishing the efficacy of nanomedicines [125]. Beyond these vesicular and tubular “highways,” resistance can be propagated through direct physical contact. Mechanical signals transmitted via enhanced intercellular contractile forces can activate pathways such as Notch signaling. This activation, in turn, upregulates the major vault protein to promote drug export from the nucleus, directly reducing chemosensitivity [126]. The significance of this interconnected network is magnified within specialized anatomical niches. In brain metastases, for example, gap junctions formed between tumor cells and resident astrocytes create direct channels for the efflux of chemotherapeutics and the exchange of pro-survival signals, thereby creating a formidable sanctuary for resistance [127].

In summary, drug resistance evolves beyond a cell-autonomous trait through a sophisticated communication infrastructure. The integrated network of exosomes, TNTs, and direct intercellular connections enables the “horizontal transmission” of MDR, transforming it into a collective, adaptable, and microenvironment-modulated phenotype within the tumor ecosystem.

### Therapy-induced dynamic remodeling of the TME

Conventional and emerging cancer therapies—including chemotherapy, radiotherapy, and immunotherapy—frequently function as powerful sculptors of the MDR landscape. Beyond merely selecting for pre-existing resistant clones, these treatments actively and systematically remodel the entire tumor ecosystem. This remodeling is initiated by therapy-induced stress responses within the tumor cells themselves. Chemotherapy, for example, can trigger non-canonical signaling pathways. One specific mechanism involves the nuclear translocation of phosphorylated Toll-like receptor 3 (TLR3). Once in the nucleus, it epigenetically reprograms pro-metastatic and pro-survival gene networks, directly enhancing cellular traits associated with metastasis and drug resistance [128].

Concurrently, the widespread cell death and damage inflicted by chemotherapy release a cascade of damage-associated molecular patterns. A key consequence is the recruitment and activation of neutrophils, leading to the formation of neutrophil extracellular traps (NETs). These NETs can subsequently activate latent transforming growth factor-β (TGF-β) within the TME, thereby driving EMT and fostering a therapy-resistant state [129]. Furthermore, certain chemotherapeutic agents may paradoxically undermine their own potential immunogenic effects. For instance, they can promote the lysosomal degradation of cyclic GMP–AMP synthase (cGAS), thus suppressing the critical immunostimulatory cGAS–STING pathway and blunting anti-tumor immunity [130]; Radiotherapy exhibits a similar dualistic impact. While capable of activating anti-tumor immunity, it can also induce tumor cells to secrete soluble factors such as amphiregulin (AREG). This secretion reprograms local myeloid populations and upregulates “don’t eat me” signals (e.g., CD47) on tumor cells, inadvertently promoting distant metastasis and immune evasion [131, 132], Consequently, the complex and dynamic state of the post-radiation immune microenvironment is a critical determinant of therapeutic efficacy versus the emergence of resistance [133].

A central outcome of this therapy-driven remodeling is the consolidation of a profoundly immunosuppressive niche. CAFs are pivotal architects of this niche. Their therapy-induced metabolic reprogramming—such as the overexpression of nicotinamide N-methyltransferase (NNMT)—can deplete essential metabolic cofactors. This depletion alters the cellular epigenetic landscape, which in turn dysregulates immune-modulatory pathways like the complement system. The net result is the systemic impairment of CD8⁺ T cell function [134]. In parallel, the phenotype and function of myeloid immune cells within the TME are profoundly skewed. In contexts responsive to immune checkpoint blockade, activated T cells can successfully “re-educate” TAMs, converting them from an immunosuppressive to a more immunostimulatory state. In resistant settings, however, this re-educative crosstalk fails, leaving macrophages locked in a suppressive phenotype that perpetuates resistance [135]. Clinical evidence further underscores the deterministic role of the baseline TME state. In non-small cell lung cancer patients treated with chemoradiotherapy, for example, resistance to subsequent consolidative anti–PD-L1 therapy is strongly associated with a pre-treatment microenvironment characterized by poor CD8⁺ T cell infiltration and high tumor cell expression of the immunosuppressive ectoenzyme CD73 [136]. In summary, therapy-induced MDR represents a continuous process of ecological evolution within the tumor.

### Bidirectional interaction and co-evolution of tumor cells and the TME

**Fig. 4:**
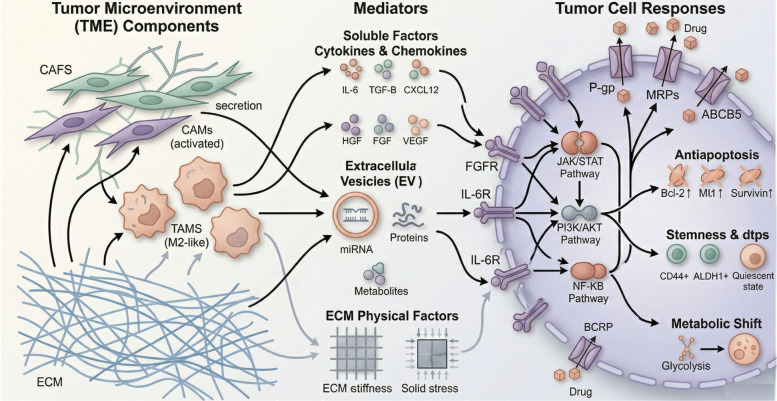
The TME drives non-cell-autonomous MDR.​ The tumor microenvironment orchestrates non‑cell‑autonomous multidrug resistance via stromal cell secretomes, extracellular matrix remodeling, hypoxic and acidic niches, and immunosuppressive networks that shield cancer cells from therapeutic insult. (CAF: Cancer-associated fibroblast; ECM: Extracellular matrix; TAM: Tumor-associated macrophage; VEGF: Vascular endothelial growth factor; TGF-β: Transforming growth factor-β; IL-6: Interleukin-6; CXCL12: C-X-C motif chemokine ligand 12; EV: Extracellular vesicle; P-gp: P-glycoprotein; MRPs: Multidrug Resistance-associated Proteins; ABCB5: ATP-binding cassette subfamily B member 5; BCRP: Breast Cancer Resistance Protein; HGF: Hepatocyte Growth Factor; FGF: Fibroblast Growth Factor)

In summary, tumor MDR emerges from the continuous, dynamic, and bidirectional co-evolution of cancer cells with their surrounding TME (Fig. 4). This partnership positions tumor cells as active orchestrators or “command centers.” They recruit, instruct, and reprogram stromal and immune cells within the TME—for example, by secreting alarmins like S100A8 to educate CAFs or releasing factors such as AREG to skew myeloid cell function—thereby proactively engineering a protective, pro-survival niche. In turn, these activated TME components provide indispensable feedback, systematically entrenching the resistant phenotype. They achieve this by constructing dense physical barriers, secreting resistance-laden exosomes, supplying metabolic sustenance, and enforcing local immunosuppression. Critically, therapeutic intervention is not merely a passive selector but a potent driver of this co-evolutionary process. It applies selective pressure that enriches for pre-resistant clones while simultaneously triggering and accelerating the remodeling of the entire TME into a state of profound immunosuppression and heightened adaptability. Therefore, the tumor and its microenvironment constitute an inseparable, co-adaptive functional unit. The essence of MDR is this very unit's systemic adaptive state—an emergent property of the tumor-TME ecosystem. Forged under therapeutic pressure through relentless bidirectional crosstalk and functional synergy, this adaptive state ultimately secures tumor survival and fuels its evolution.

## Clinical challenges and translational gaps in tumor MDR

### Clinical monitoring and early warning

**Fig. 5:**
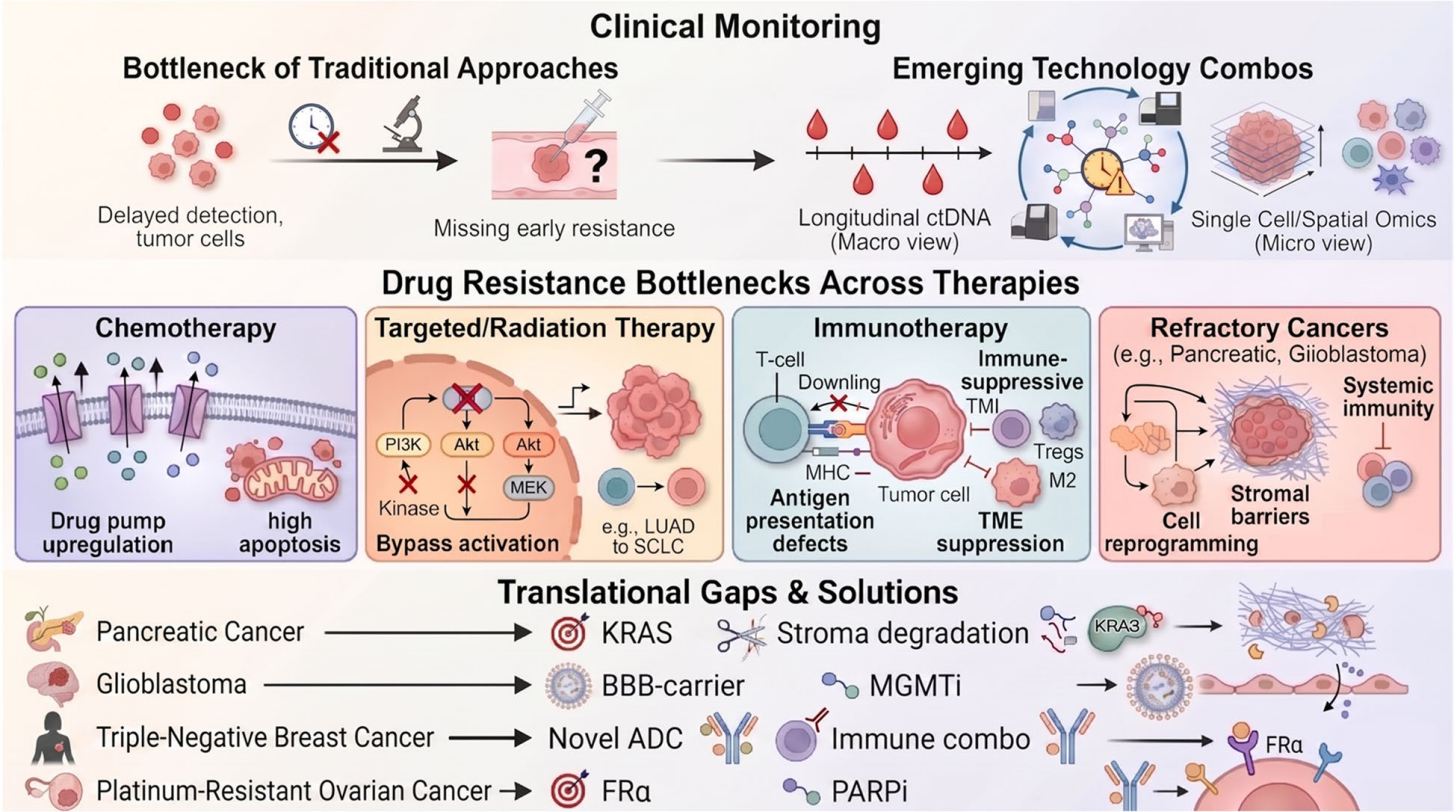
Clinical bottlenecks, mechanistic landscapes, and therapeutic solutions in tumor MDR. Multidrug resistance manifests as a convergence of pharmacokinetic failure, tumor heterogeneity, and dynamic adaptive evolution, necessitating precision combinatorial strategies spanning molecularly targeted agents, immunomodulation, and advanced drug delivery technologies. (MGMTi: O⁶-methylguanine-DNA methyltransferase inhibitor; PARPi: Poly polymerase inhibitor; KRAS: Kirsten rat sarcoma viral oncogene homolog; SCLC: Small cell lung cancer; LUAD: Lung adenocarcinoma)

The clinical management of tumor MDR is profoundly complicated by tumor clonal evolution and spatiotemporal heterogeneity. Conventional strategies reliant on lagging imaging and static tissue biopsies often miss the critical window for early intervention (Fig. 5). This limitation has propelled the dynamic monitoring of minimal residual disease (MRD) to the forefront as a pivotal strategy for early detection. Among MRD detection modalities, liquid biopsy leveraging circulating tumor DNA (ctDNA) has become a cornerstone of modern clinical decision-making, primarily due to its unique ability to overcome spatial sampling bias and provide a systemic molecular snapshot of the tumor burden [137, 138].

Ystematic longitudinal tracking of ctDNA offers a powerful macroscopic perspective. It can capture the evolution of micro-metastases and immunotherapy-related clonal dynamics [139, 140], and has proven valuable for the early prediction of resistant relapse across diverse malignancies, including acute myeloid leukemia [141], colorectal cancer [142] and breast cancer exhibiting resistance to neoadjuvant therapy [143]. Advances in detection sensitivity are further refining this approach. Personalized, tumor-informed ctDNA assays enable more precise molecular residual disease stratification in solid tumors [144], This refined stratification creates actionable windows for therapeutic adaptation, guiding interventions such as consolidative immunotherapy in small cell lung cancer [145] and enabling dynamic, risk-adaptive strategies in nasopharyngeal carcinoma [146].

However, the macroscopic, population-averaged nature of liquid biopsy can obscure crucial biological events occurring at the single-cell level, such as the emergence of rare resistant phenotypes and intricate cell–microenvironment interactions. To illuminate this “microscopic blind spot,” cutting-edge analytical technologies are being deployed. These include single-cell bioanalyzers for the real-time quantification of multidrug efflux pump activity in individual cells [147] and time-resolved methods to track extracellular vesicle secretion dynamics from single cells, assessing resistance propagation [148]; In parallel, spatial transcriptomics provides an unprecedented view, enabling the in situdissection of how the TME is remodeled to foster a drug-tolerant ecosystem [149].

In summary, the integration of systematic, longitudinal tracking of ctDNA with high-resolution single-cell and spatial omics analyses has begun to construct a multi-dimensional early-warning network for MDR [150].

### Clinical dilemmas of MDR across different therapies

The clinical dilemma of tumor MDR stems from the profound complexity of tumor biology, characterized by high cellular heterogeneity and dynamic adaptability in response to diverse therapeutic selective pressures. Although different treatment modalities employ distinct mechanisms of action, tumor cells frequently converge on a resistance strategies by activating pivotal survival signals and remodeling both their intrinsic state and the surrounding microenvironment.

Cytotoxic Chemotherapy.​ The enduring challenge for classical chemotherapeutics centers on enhanced drug efflux and elevated apoptotic thresholds. A key driver of this phenotype is the sustained activation of master stress-response regulators such as NF-κB. Its persistent signaling not only transcriptionally upregulates ABC drug efflux transporters but also broadly orchestrates a pro-survival and pro-inflammatory program. This program regulates anti-apoptotic proteins and shapes the TME, thereby establishing a foundation for broad-spectrum cross-resistance [151].

Targeted Therapy and Radiotherapy. While revolutionary, precision targeted agents encounter rapid and diverse adaptive resistance. In hormone receptor-positive breast cancer, for example, resistance to endocrine therapy and CDK4/6 inhibitors is commonly driven by acquired ESR1mutations, reactivation of the PI3K/AKT/mTOR pathway, and cyclin E1 overexpression [152]; Radiotherapy resistance, notably in malignancies such as glioma, is closely linked to enhanced pro-survival signaling mediated by axes including integrin ITGA2/AKT [153]. A particularly formidable challenge is therapy-induced lineage plasticity, where targeted pressure triggers profound cellular identity reprogramming. The transformation of lung adenocarcinoma to small cell lung cancer is a prime example; this histological switch fundamentally alters the tumor's biology and therapeutic vulnerabilities, representing a major cause of targeted therapy failure [154].

Immunotherapy.​ The advent of immune checkpoint inhibitors has shifted the MDR battlefield to the tumor–immune interface, revealing widespread primary and acquired immune resistance. The underlying mechanisms form a multi-tiered defensive architecture. Tumor-intrinsic adaptations include defects in antigen presentation machinery and mutations in the interferon-γ signaling pathway, which enable immune evasion. Concurrently, microenvironmental remodeling—driven by the infiltration of immunosuppressive cells (e.g., Tregs, MDSCs) and secretion of inhibitory cytokines (e.g., IL-4)—collaboratively establishes an immune- “cold” tumor state [155, 156]. A further layer of complexity is the compensatory upregulation of alternative immune checkpoints (e.g., LAG-3, TIM-3), which often renders monotherapy targeting the PD-1/PD-L1 axis ineffective [157]. The systemic and heterogeneous nature of this resistance network limits the population that derives durable benefit from current immunotherapies, as observed in non-small cell lung cancer, underscoring the urgent need for mechanism-informed combination strategies [158].

Emerging Solutions.​ Confronting these multifaceted challenges necessitates innovative tools. Liquid biopsy platforms analyzing ctDNA provide a means for the dynamic monitoring of minimal residual disease, offering potential for ultra-early resistance detection [159]. Furthermore, advanced preclinical models such as patient-derived tumor immune organoids are emerging as powerful platforms. They enable the individualized simulation of the complex TME, dissection of resistance mechanisms, and screening of rational combination therapies, thereby serving as a critical bridge from mechanistic discovery to clinical translation [160]. In summary, the distinct yet interconnected clinical dilemmas of MDR observed across major treatment classes—chemotherapy, targeted therapy, radiotherapy, and immunotherapy—collectively represent the multifaceted and adaptive evolutionary capacity of tumors.

### Clinical dilemmas of MDR in refractory tumors

The clinical management of MDR in refractory cancers—including pancreatic cancer, glioblastoma, triple-negative breast cancer (TNBC), and platinum-resistant ovarian cancer (see Table 1)—remains a formidable and largely unmet challenge, as both conventional and emerging therapeutic strategies frequently encounter failure.

**Table 1:** Characteristics and manifestations of MDR in refractory tumors

	MDR specific mechanisms	Clinical manifestations	Current treatment strategies	References
Pancreatic Cancer	Circular RNAs inhibit pyroptosis; Stromal barrier impedes penetration; KRAS mutations block apoptosis	Early pan-resistance; Rapid resistance to gemcitabine/AG; Poor immunotherapy response	Nivolumab + Ipilimumab (combined or sequential); Dosing schedule optimization; GDF-15 blockade	[161–163]
HCC	Bypass signaling activation (EGFR/FGFR); Viral-mediated MDR gene expression; Disordered vasculature limits delivery	Rapid TKI resistance; Higher risk with HBV; Aggressive recurrence after withdrawal	TKI + PD-1 blockade; Anti-angiogenics to normalize vessels; Investigational HDAC inhibitors in combination with TKIs	[164–167]
Ovarian Cancer (BRCA-mutant)	DDR network reprogramming; HR repair capacity abnormally restored; Malignant ascites-enriched exosome-mediated drug efflux	Relapse within months; Resistant peritoneal nodules; Ascites as sanctuary	PARPi maintenance; FRα-targeting ADC; HIPEC	[168–170]
Melanoma	Phenotypic transformation by BRAF inhibitors; GDF-15 exhausts T cells; Impaired antigen presentation	Rapid progression after initial response; High brain metastasis rate; Dual kinase/immunotherapy resistance	Dosing schedule optimization; GDF-15 blockade; PD-1/CTLA-4 combination therapy	[171–174]
NSCLC	Binding pocket mutations; Lineage transformation to SCLC; DTPs dormancy	Loss of target dependence; Heterogeneous responses; Insensitivity to subsequent chemo	Bispecific antibody (Amivantamab) combos; Targeted + chemo; PROTACs development	[175–177]
Breast Cancer (HER2-positive)	APOBEC3B accelerates ESR1 mutations; Metabolic shift to glycolysis; Local immune barrier	Micro-metastases post-endocrine therapy; Primary taxane resistance in TNBC; CD8 + T cell exclusion	Next-gen SERDs; ADC bystander effect; Glycolysis inhibition + chemo	[178–180]
Colorectal Cancer (BRAF V600E-mutant)	Compensatory EGFR reactivation; Antioxidant axis activation; Inflammatory microenvironment	Rapid single-target inhibitor failure; Desmoplasia; Angiogenesis inhibitor failure	EGFR antibody combos; Wnt inhibitors + chemo; Anti-inflammatory (e.g., COX2) inhibitors	[181–183]
Glioblastoma	Stemness maintenance (SOX2/OCT4); MGMT-mediated DNA repair; BBB limits delivery	Absolute TMZ/radiotherapy resistance; Near 100% recurrence; Poor drug penetration to CNS	BBB-penetrating nanocarriers; MGMT inhibitors + TMZ; Targeting stemness-associated signaling (e.g., TRIM16-OPTN axis)	[174, 184–186]

*HCC* Hepatocellular Carcinoma, *HR* Homologous recombination, *ADC* Antibody–drug conjugate, *TKI* Tyrosine kinase inhibitor, *EGFR* Epidermal growth factor receptor, *FGFR* Fibroblast growth factor receptor, *GDF-15* Growth differentiation factor 15, *NSCLC* Non-small cell lung cancer, *SCLC* Small cell lung cancer, *PROTACs* Proteolysis-targeting chimeras, *TNBC* Triple-negative breast cancer

Cell-intrinsic Reprogramming. A primary driver of resistance in these cancers is the acquisition of core survival advantages through multi-layered intrinsic adaptations. In pancreatic cancer, for instance, the circular RNA *circERC1*promotes chemotherapy resistance by simultaneously inhibiting pyroptosis and remodeling the ECM [187]; TNBC, in contrast, often develops resistance to neoadjuvant chemotherapy via a pronounced metabolic reprogramming that favors glycolysis [188]. Similarly, in glioblastoma, chemoresistance to temozolomide is directly mediated by the LMNA-PRKDC axis, which enhances DNA repair capacity [189].

Microenvironmental Remodeling. Beyond cell-autonomous changes, tumor cells actively reshape their surrounding niche to construct dual physical and biochemical barriers. Pancreatic cancer cells, for example, can “educate” and activate CAFs by delivering the oncogenic protein KRASG12Dto them via EVs, thereby indirectly bolstering the tumor's own chemotherapy resistance [190]. In glioblastoma, resistance to temozolomide is associated with the upregulation of P4HA1. This enzyme activates YAP signaling through prolyl hydroxylation, driving excessive collagen synthesis and the formation of a dense, armor-like ECM that physically impedes drug penetration [191].

Systemic Immunosuppression. Perhaps the most complex challenge is the profound and pervasive immunosuppressive state characteristic of these malignancies. Glioblastoma is a quintessential “immune-cold” tumor, with a microenvironment dominated by immunosuppressive myeloid cells that severely exhaust and exclude cytotoxic T cells, creating a major barrier to immunotherapy [192]. In TNBC, immune evasion mechanisms are remarkably sophisticated. Tumor cells can polarize macrophages toward an immunosuppressive M2 phenotype by activating CSF1 through the epigenetic regulator MORF4L2 [193]; Alternatively, they can dually suppress antigen presentation at transcriptional and signaling levels by overexpressing FAM114A1 [194].

Emerging Therapeutic Avenues. Despite these hurdles, clinical translational research is identifying promising new strategies. The Phase III success of the folate receptor alpha (FRα)-targeting antibody–drug conjugate (ADC) mirvetuximab soravtansine in platinum-resistant ovarian cancer provides a first-in-class option that significantly improves overall survival for a population with historically few effective therapies [168]. For immunotherapy-resistant TNBC, novel combination strategies aimed at “mobilizing” intratumoral antigen-presenting mast cells are under investigation [195], Furthermore, mechanistic explorations, such as targeting the TRIM16-OPTN axis with the natural compound sanggenol L to reverse glioma chemoresistance, highlight the potential of mechanism-informed interventions [196]. In summary, MDR in refractory cancers epitomizes the extreme adaptive and evolutionary capacity of tumors. Addressing their profound unmet clinical need necessitates a fundamental paradigm shift—from a reductionist strategy focused on “one-dimensional killing” to an integrative approach dedicated to the “multi-dimensional remodeling” of the entire tumor ecosystem.

## Reversal strategies for tumor MDR

Given the complexity of MDR mechanisms, overcoming resistance requires shifting from singular-target inhibition to multidimensional intervention strategies. Effective clinical translation demands simultaneous targeting of tumor-intrinsic vulnerabilities and dismantling of the protective, immunosuppressive TME. This section reviews these evolving reversal strategies, beginning with direct modulation of cell death and epigenetic pathways. We then focus on TME reprogramming to convert “cold” tumors into “hot” ones. Furthermore, we highlight emerging technologies—such as artificial intelligence, single-cell multi-omics, and nanomedicine—that enable intelligent, synergistic regimens. Collectively, these approaches aim to establish a closed-loop clinical framework integrating dynamic monitoring with adaptive intervention.

### Targeting intrinsic drug resistance mechanisms in tumors

**Fig. 6:**
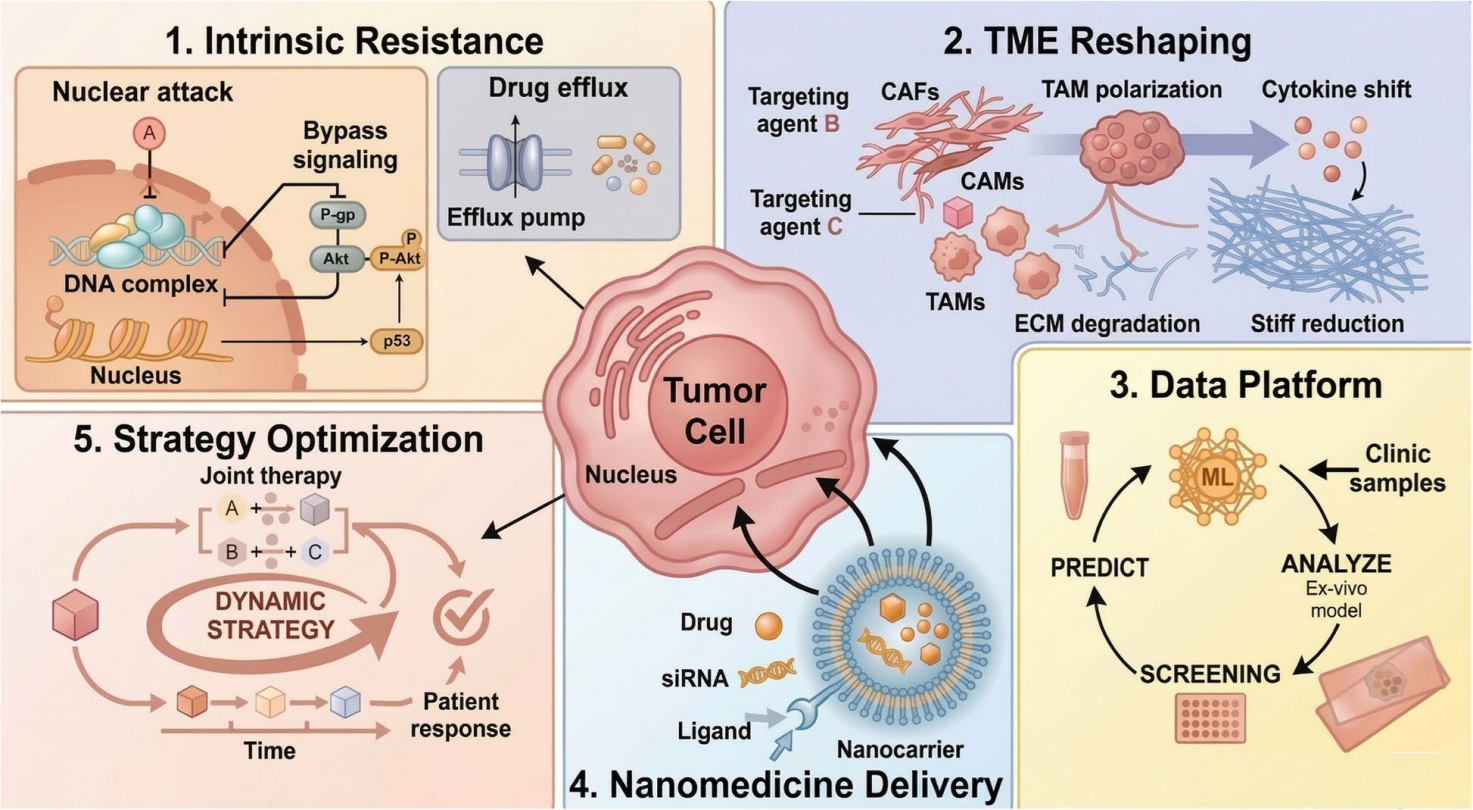
Multi-dimensional strategy framework for overcoming tumor MDR. A rationally integrated intervention framework addresses tumor multidrug resistance across molecular, cellular, and systemic levels through targeted pathway inhibition, microenvironment reprogramming, next-generation drug delivery platforms, and precision combinatorial regimens

Targeting the cell-intrinsic mechanisms that underpin drug resistance is a potent strategy for reversing tumor MDR (Fig. 6). This approach encompasses several key avenues: Inducing Novel Cell Death Pathways. A promising frontier involves activating non-apoptotic programmed cell death, such as ferroptosis and cuproptosis, to bypass classical apoptosis resistance. Resistant cells often develop specific metabolic dependencies to evade these pathways. For instance, tumor-repopulating cells confer structural resistance to ferroptosis by downregulating PCK2, thereby reducing the synthesis of peroxidation-susceptible membrane lipids [197]; Conversely, cancers like KRAS-mutant lung adenocarcinoma become addicted to the ferroptosis suppressor protein FSP1; targeting FSP1 selectively triggers ferroptosis in these cells [198]. Importantly, these death pathways can be harnessed to synergize with and resensitize tumors to conventional therapies. Radiotherapy can induce cuproptosis by upregulating copper transporters, and its combination with copper ionophores effectively overcomes radioresistance [199]; Similarly, in BRAF^(V600E) colorectal cancer, where resistance to BRAF/EGFR inhibitors is driven by GPX4 upregulation, targeting the upstream kinase PLK1 triggers ferroptosis and reverses this resistance [200].

Disrupting DNA Repair and Epigenetic Adaptation. Precisely targeting the DNA damage repair and epigenetic networks that sustain resistant cells is another cornerstone strategy. In osteosarcoma, the kinase PKMYT1 drives cisplatin resistance by phosphorylating NPM1 and impeding the recruitment of DNA repair factors [201]; In colorectal cancer, the USP10/XAB2/ANXA2 axis promotes oxaliplatin resistance by enhancing DNA repair fidelity [202]. Beyond direct repair, targeting the epigenetic machinery can reverse deeply entrenched resistant phenotypes. Inhibiting the histone methyltransferase NSD2 reverses neuroendocrine differentiation and associated therapy resistance in prostate cancer [203]; The DNA methyltransferase DNMT1 can also stabilize replication forks via a non-catalytic function, and its inhibition reverses resistance to PARP inhibitors [204].

Re-engineering Drug Efflux and Sequestration. Efforts to counter the classic ABC transporter-mediated efflux are also evolving. Innovative strategies include drug repurposing. The proton pump inhibitor lansoprazole, for example, acts as a competitive substrate for ABCB1/G2 transporters and impedes lysosomal drug sequestration, effectively reversing MDR at non-toxic doses [205].

In summary, successfully reversing intrinsic MDR hinges on a multi-pronged approach: identifying the unique vulnerabilities acquired by drug-resistant cells and launching coordinated attacks against these adaptive networks. This strategy is fundamental for achieving a durable breakthrough against resistant disease.

### Targeting tumors TME

Under sustained therapeutic pressure, highly heterogeneous solid tumors actively remodel their surrounding physical and immune landscape, constructing a formidable defensive architecture. Consequently, strategies that target the TME itself have gained prominence as a cutting-edge approach for reversing MDR. This paradigm centers on two interdependent objectives: dismantling the physical and metabolic barriers orchestrated by CAFs and reprogramming the immunosuppressive networks dominated by myeloid cells.

The therapeutic strategy against CAFs has evolved from non-specific depletion to the precise reversal of their pro-tumor, therapy-resistant state. Tumor cells can aberrantly activate and “educate” CAFs, for instance, by secreting alarmins like S100A8 or delivering EVs loaded with oncoproteins such as KRASG12D. This reprogramming transforms CAFs into active accomplices that drive chemotherapy resistance and promote pathological stromal remodeling [98, 190]. Once activated, specific CAF subsets—such as those expressing TSPAN8—construct a composite fortress. Through metabolic reprogramming, suppression of tumor cell pyroptosis, and the copious secretion of collagen and exosomes (e.g., containing CCT6A), they simultaneously impede drug penetration and enhance cancer stemness [103, 206]. New therapeutic modalities are therefore designed to “de-activate” these CAFs. Promising approaches include using nanocarriers to deliver vitamin D receptor agonists to induce CAF quiescence or targeting signaling nodes like connective tissue growth factor (CTGF) to block their aberrant activation, thereby breaking down the stromal barrier and resensitizing tumors to therapy [207, 208].

In parallel, disrupting the immunosuppressive network anchored by TAMs is critical for overcoming immunotherapy resistance. Therapeutic pressure drives the adaptive evolution of myeloid cells within the TME, leading to the expansion of highly immunosuppressive subsets such as SPP1hiTAMs. These cells directly contribute to CD8⁺ T cell exhaustion via mechanisms like adenosine signaling [209]. Counterstrategies are advancing on two fronts: metabolic intervention and intelligent reprogramming. Targeting metabolic regulators—such as antagonizing estrogen-related receptor alpha (ERRα) or inhibiting phospholipid transfer protein (PLTP)—can modulate myeloid cell function, reduce M2-like TAM infiltration, and thereby sensitize tumors to immunotherapy [210, 211]. More innovatively, “smart” therapies aim to re-educate TAMs. One strategy involves engineering immunocytokines that target the inhibitory receptor TREM2 and locally release IL-2, simultaneously blocking suppression and activating T/NK cells. Another approach targets the Wnt pathway co-activator BCL9 within tumor cells, which in turn promotes a shift in macrophages toward an antigen-presenting phenotype, synergistically overcoming immune resistance [212, 213].

In summary, successfully reversing MDR by targeting the TME requires a refined understanding of its intricate cellular crosstalk. The path forward lies in developing integrated, combination strategies that can sequentially and synergistically dismantle the physical barriers erected by CAFs while reprogramming the immunosuppressive circuits maintained by myeloid cells.

### Emerging technologies and precision therapy for MDR

The paradigm for precision therapeutic strategies against tumor MDR is undergoing a profound revolution, driven by the convergence of emerging technologies such as single-cell multi-omics, artificial intelligence (AI), and high-throughput functional screening. Within this revolution, AI and machine learning play a central, integrative role by synthesizing multi-source data to generate predictive and actionable insights. Dedicated computational foundation models, exemplified by tools like CancerGPT which are trained on vast single-cell transcriptomic datasets, provide powerful frameworks for deciphering the complex mechanisms underlying drug resistance [214]. More directly relevant to clinical translation are intelligent computational pipelines such as PERCEPTION, which utilize transfer learning to integrate a patient's unique single-cell tumor profile with extensive drug screening databases. This capability enables the prediction of not only initial therapeutic efficacy but also the potential emergence of resistant clones *prior to*treatment, offering critical insights for the design of pre-emptive, personalized combination strategies [215]. This predictive power is further augmented through integration with complementary multimodal data—including genomic alterations and histopathological images—which refines patient risk stratification and identifies putative resistance pathways, thereby completing a robust strategic cycle of Prediction​ [216].

Building upon these predictive insights, high-throughput functional genomics and spatial omics technologies provide unprecedented resolution for the in-depth Analysis​ of resistance mechanisms. Large-scale, systematic CRISPR screening campaigns are mapping the comprehensive genetic networks that confer resistance to multiple chemotherapeutic agents. A key revelation from such efforts is that evolutionarily distinct resistance types may converge upon shared molecular vulnerabilities, such as PLK4, pointing the way toward novel, broad-spectrum strategies to reverse resistance [217]. Concurrently, single-cell and spatial transcriptomic analyses elevate the analytical resolution to the level of individual cellular lineages and their spatial context. They enable the precise identification of key cellular subpopulations—for instance, NPC-like cell clusters with high *MAZ*expression in glioma—and the specialized ecological niches that drive recurrence and resistance, thereby elucidating the fundamental cellular and architectural foundations of MDR development [218].

Furthermore, novel intervention tools and high-fidelity experimental models constitute the final, crucial Intervention​ and validation loop in this pipeline. For historically “undruggable” targets implicated in resistance, protein degradation technologies like proteolysis-targeting chimeras (PROTACs) offer a revolutionary modality by inducing the ubiquitin-dependent degradation of specific target proteins to overcome resistance [219]. The validation of these and other predicted strategies relies critically on advanced preclinical models. Innovative platforms, such as those combining 3D bioprinting with label-free imaging, now permit high-throughput, real-time, and dynamic assessment of drug efficacy at single-organoid resolution, significantly enhancing the predictive value and translational efficiency of preclinical research [220]. In summary, emerging technologies are synergistically empowering the entire continuum of Prediction–Analysis–Intervention. This integrated, closed-loop approach is systematically propelling MDR therapy into a new era defined by precision, predictability, and proactive intervention.

### Emerging biological agents and epitranscriptomic strategies in MDR reversal

Strategies for reversing tumor MDR are undergoing a paradigm shift—from a singular focus on ABC transporter inhibition toward the modulation of multilayered regulatory networks. Among these, the convergence of epitranscriptomic interventions and next-generation biologics represents an area of exceptional translational potential. At the epitranscriptomic level, dynamic dysregulation of N⁶-methyladenosine (m6A) modification has emerged as a core upstream driver of chemoresistance and CSC stemness. Specifically, the m6A reader YTHDF1 promotes NOTCH1 translation to activate stemness programs in colorectal cancer, whereas YTHDF3 enhances DNA damage repair gene expression in glioblastoma through recognition of METTL3-dependent m6A modifications. Conversely, pharmacological targeting of the m6A demethylase ALKBH5 effectively suppresses CSC phenotypes and restores chemosensitivity, underscoring the therapeutic promise of intervening across the entire m6A writer–eraser–reader axis [221–224]. Complementing these molecular approaches, next-generation biologics circumvent canonical resistance mechanisms through distinct yet compatible strategies. Bispecific T-cell engagers, such as DLL3-targeted Tarlatamab, recruit cytotoxic T cells to eliminate chemorefractory small-cell lung cancer, whereas bispecific antibodies and dual-target nanobody–drug conjugates bypass resistance mediated by receptor downregulation and endocytic escape via multipronged antigen engagement [225–229]. Furthermore, rational combination regimens—exemplified by DR5-targeted antibody–drug conjugates paired with CDK inhibitors—have demonstrated efficacy in overcoming intrinsic resistance in advanced colorectal cancer [230]. Collectively, these findings suggest that integrating epitranscriptomic modulators with engineered biologics can coordinately dismantle both CSC-driven persistence and drug uptake/target loss barriers, thereby establishing a compelling framework for advancing MDR reversal strategies toward clinical application.

### Nanomedicine and smart delivery systems

#### Integrative nanoplatforms toward theranostics

Nanomedicine-based strategies are emerging as a powerful arsenal to dismantle the complex network of tumor MDR. This is achieved through the intelligent design of nanocarriers that systematically integrate three complementary pillars: achieving precision delivery​ to resistant cells and niches, directly intervening in critical survival pathways​ like metabolism and cell death, and actively remodeling the immunosuppressive TME.

Precision Delivery.​ The first pillar focuses on engineering nanocarriers for targeted, deep-tissue drug delivery. Sophisticated functionalization enables these carriers to overcome formidable physiological barriers. A prime example is the dual challenge posed by the blood–brain barrier and the dense extracellular matrix in glioblastoma. Here, biomimetic nanocarriers (e.g., camouflaged with microglial cell membranes) and dual-responsive systems (e.g., sensitive to tumor-associated MMP2 enzymes and the acidic pH) significantly enhance accumulation and penetration at the brain tumor site, laying the groundwork for reversing CNS drug resistance [231, 232]. Beyond merely crossing barriers, active targeting leverages unique molecular signatures on resistant cells or within the tumor stroma—such as overexpressed folate receptors or integrins—to precisely home nanomedicines to the lesion, dramatically improving local drug bioavailability [233].

Multi-Mechanistic Intervention.​ The second pillar exploits nanoplatforms as ideal vehicles for the co-delivery of diverse therapeutic agents, enabling a coordinated, multi-pronged attack on core resistance mechanisms. A foundational strategy is the co-encapsulation of chemotherapeutics with P-glycoprotein inhibitors or silencing RNAs, which simultaneously increases intracellular drug concentration and disables the primary efflux pumps responsible for MDR [234]; Nanocarriers are also uniquely suited to bypass apoptotic resistance by synergistically inducing alternative cell death pathways. This can involve leveraging the intrinsic properties of nanomaterials (e.g., MXenes or noble metal nanoshells for photothermal therapy) or co-delivering inducers to trigger ferroptosis, cuproptosis, or immunogenic cell death [235, 236]; Targeting metabolic reprogramming to disrupt the tumor cell's “metabolic armor” is a key strategy. This includes, for example, using mitochondrial-targeted nanoprodrugs to inhibit both aerobic glycolysis and oxidative phosphorylation, thereby reversing cisplatin resistance [237], or delivering glycolysis inhibitors to deprive drug-resistant cells of their energy source [238].

Microenvironment Remodeling.​ The third pillar aims to reverse the non-autonomous aspects of MDR by using nanomedicines to reprogram the tumor immune landscape, converting immunologically “cold” tumors into “hot,” therapy-responsive ones. Catalytic nanomaterials, such as gold–platinum bimetallic nanoshells, can alleviate tumor hypoxia by decomposing endogenous H₂O₂, thereby indirectly revitalizing immune cell function [239]; Other nanoformulations, like certain ruthenium-based complexes, can induce immunogenic cell death and modulate neutrophil extracellular traps to systemically activate anti-tumor immunity [240]. The most advanced approaches seek to directly “re-educate” immunosuppressive cells; for example, by targeting and repolarizing TAMs or by degrading key immunosuppressive proteins within the niche [241].

In summary, nanomedicine is driving the evolution of MDR reversal strategies toward greater intelligence, integration, and personalization. The trajectory points toward the development of closed-loop, adaptive “nano-theranostic” systems. These future platforms would integrate the functions of perception​ (dynamic monitoring of resistance biomarkers), judgment​ (AI-informed decision-making), and execution​ (triggered, precise drug release), ultimately realizing a pre-emptive and self-adjusting therapeutic paradigm against MDR [242].

#### Synergistic reversal strategies based on nanocarriers

The evolution of precision medicine has fundamentally reshaped the therapeutic approach to tumor MDR, shifting the paradigm from isolated pharmacologic inhibition toward a systematic strategy of network dismantling. This advanced paradigm relies on the coordinated synergy of multiple therapeutic mechanisms, for which functionalized nanocarriers serve as an indispensable integration platform. Their pivotal role stems from an inherent multifunctionality that enables the spatiotemporally controlled co-delivery of diverse therapeutic agents—including conventional chemotherapeutics, physical energy-based modalities, and gene-regulatory elements—thereby orchestrating a concerted “combo strike” against the intricate tumor resistance network.

A cornerstone of this multi-pronged attack is physical energy intervention, exemplified by photodynamic therapy (PDT) and photothermal therapy (PTT). These modalities are uniquely advantageous because their mechanisms of action bypass passive drug-uptake pathways. PDT operates via photosensitizers that, upon light activation, generate cytotoxic reactive oxygen species (ROS) to induce direct cell killing and immunogenic cell death, an effect impervious to classical drug efflux pumps. PTT, conversely, employs photothermal conversion to generate localized hyperthermia, achieving tumor ablation through thermal damage that can also compromise cell membrane integrity and protein function [243–245]. The therapeutic potential of these approaches is profoundly amplified through nanoengineering. By co-encapsulating PDT/PTT agents (photosensitizers or photothermal materials) with chemotherapeutic drugs within a single nanocarrier, a powerful synergistic effect can be unlocked. Specific light irradiation then triggers not only the inherent phototoxic effects but also controlled drug release and potent immunogenic activation, forging a novel pathway to overcome both apoptosis resistance and microenvironment-mediated immunosuppression [246, 247].

Simultaneously, achieving durable reversal of MDR necessitates the precision targeting of its genetic foundations. Nucleic acid therapeutics—such as small interfering RNA (siRNA) and antisense oligonucleotides (ASOs)—and gene-editing tools like the CRISPR/Cas9 system have emerged as powerful instruments for this purpose. They can be designed to specifically silence or knockout the expression of core resistance drivers, including drug efflux pumps, pro-survival signaling proteins, and DNA repair factors [248, 249]. The clinical translation of these biomacromolecules, however, is hindered by substantial delivery challenges pertaining to poor in vivo stability, lack of tumor specificity, and inefficient cellular internalization. Nanocarrier systems—including liposomes, polymeric nanoparticles, and exosomes—provide a robust solution. By encapsulating the payload, prolonging systemic circulation, and incorporating surface functionalization (e.g., with targeting ligands), these systems dramatically enhance the tumor-specific accumulation and intracellular delivery efficiency of nucleic acid drugs [250, 251], This advanced delivery capability makes it feasible to precisely “switch off” resistance genes at the transcriptional or translational level, a principle convincingly demonstrated by studies in which functionalized nanoparticles successfully reversed tumor drug resistance through targeted gene silencing [252, 253].

The convergence of these advancements points toward the most sophisticated frontier: the construction of intelligent, “all-in-one” nano-theranostic platforms. Such a platform could, for instance, integrate within a single nanoparticle a chemotherapeutic drug, an siRNA targeting a key resistance gene, and a photosensitizer for adjunctive therapy [254]. Engineered to respond to external or internal stimuli—such as light, the specific pH of the TME, or overexpressed enzymes—this system would achieve on-demand drug release and the synchronized activation of a multi-pronged attack. This includes direct cytotoxicity from the chemotherapy, suppression of cellular defense mechanisms by the nucleic acid drug, and additional physical killing coupled with immune microenvironment remodeling initiated by phototherapy [255, 256]. This multimodal, synergistically orchestrated intervention strategy represents the vanguard in the battle against complex MDR networks. Its ultimate success will hinge on the meticulous design of nanocarrier properties and a profound, mechanistic understanding of the optimal spatiotemporal relationships between the co-delivered therapeutic agents.

### Optimization of combination therapy strategies

The clinical management of MDR is undergoing a paradigm shift, evolving from interventions against single molecular targets towards integrated, multi-dimensional strategies powered by novel technologies. This progression encompasses advances in dynamic monitoring, mechanism-informed therapeutic intervention, and the strategic sequencing of advanced agents. The ability to dynamically track the emergence of resistance has been transformed by non-invasive molecular imaging, which overcomes the spatial and temporal limitations of serial invasive biopsies. A key example is the use of radionuclide imaging agents that target immune checkpoints such as LAG-3 and PD-L1. This technology enables the longitudinal, in vivo visualization of tumor immune microenvironment evolution, providing critical insights into the kinetics of immunotherapy resistance [257]. Simultaneously, therapeutic modalities are being redesigned to attack resistance through non-canonical pathways. Innovations in radiotherapy, such as hybrid carbon-ion and photon therapy, bypass traditional apoptotic resistance mechanisms. In glioblastoma, this approach can precisely induce NCOA4-mediated ferritinophagy, triggering ferroptosis to directly dismantle the tumor's robust defensive barrier [258]. Parallel progress in biologic therapeutics is deepening responses against established resistance. For non-small cell lung cancer (NSCLC) resistant to both third-generation EGFR tyrosine kinase inhibitors and chemotherapy, the dual blockade strategy combining the bispecific antibody amivantamab with the EGFR-TKI lazertinib demonstrates significant potential for reversing profound MDR [259]; Furthermore, the strategic use of targeted agents as maintenance therapy following initial chemo-radiotherapy is emerging as a key tactic to preempt the expansion of resistant clones [260]. Substantive inroads are also being made against resistance to immune checkpoint inhibitors. Promising strategies include biomarker-guided, dual-immunotherapy combinations selected based on features like high tumor immunogenicity [261], Another approach involves the systemic remodeling of the immunosuppressive niche, for instance by neutralizing key microenvironmental factors such as GDF-15 to reinvigorate antitumor immunity [262], In summary, these converging technological advances—spanning precise monitoring, mechanism-informed therapy, and intelligent therapeutic sequencing—collectively provide the tools to systematically deconstruct the integrated biological networks that sustain MDR.

## Conclusion and perspective

The conceptual framework of cancer MDR has definitively shifted from a static “drug efflux” model to a dynamic, multidimensional defense ecosystem. As synthesized herein, MDR is an emergent property of bidirectional tumor-TME crosstalk, integrating cell-intrinsic plasticity (e.g., CSC/DTP dynamics, metabolic-epigenetic rewiring) with extrinsic non-autonomous defenses (e.g., CAF-mediated desmoplasia, immune exclusion). Conventional therapeutic failure stems fundamentally from targeting isolated nodes within this network, overlooking the functional redundancy that facilitates tumor cell switching between transient adaptive tolerance and fixed genetic resistance along the “resistance continuum.”

This dual-layered architecture​ evolves spatiotemporally: temporally via evolutionary subtypes (de novo to cross-resistance) securing clonal selection windows, and spatially through TME-facilitated horizontal propagation​ of resistance (e.g., TNT-mediated mitochondrial transfer). Crucially, this adaptive reprogramming exposes actionable vulnerabilities. The reliance of DTPs on H4K20me3-mediated epigenetic silencing​ and the metabolic addiction of resistant clones to OXPHOS​ represent high-value therapeutic targets, necessitating a strategic pivot from “mono-targeted killing” to “multidimensional ecological remodeling.”

Future strategies must converge on the integration of predictive and interventional technologies. Leveraging artificial intelligence, single-cell multi-omics, and longitudinal liquid biopsies will enable dynamic early-warning networks capable of prospecting resistance trajectories. These predictive capabilities must be paired with next-generation interventions—including spatiotemporally controlled nanomedicine, PROTACs, and immunotherapies targeting CSC/DTP-specific antigens (e.g., TROP2)—to synergistically dismantle the tumor's intrinsic survival fortress and extrinsic physical-immune barriers.

Realizing this vision requires establishing a closed-loop, adaptive clinical framework. This entails replacing static protocols with dynamic adjustments guided by real-time molecular surveillance and adopting multidimensional endpoints that assess ecosystem remodeling (stromal reorganization, immune infiltration) alongside tumor regression. Although formidable challenges persist in navigating tumor heterogeneity, a systems-biology approach integrating mechanistic dissection with interdisciplinary innovation offers the most rational path to transform cancer from a lethal disease into a manageable chronic condition.

## Data Availability

This review article does not contain any new primary data. All data supporting the findings of this study are derived from previously published articles, which are cited in the reference list.
